# Phytochemical Study on Antioxidant and Antiproliferative Activities of Moroccan *Caralluma europaea* Extract and Its Bioactive Compound Classes

**DOI:** 10.1155/2020/8409718

**Published:** 2020-03-19

**Authors:** Fatima ez-zahra Amrati, Mohammed Bourhia, Meryem Slighoua, Samir Ibnemoussa, Ahmed Bari, Riaz Ullah, Amal Amaghnouje, Francesca Di Cristo, Mohammed El Mzibri, Anna Calarco, Laila Benbacer, Dalila Bousta

**Affiliations:** ^1^Laboratory of Neuroendocrinology, Nutritional and Climatic Environment, Faculty of Sciences Dhar El Mahraz, Sidi Mohamed Ben Abdellah University, Fez 30000, Morocco; ^2^Laboratory of Chemistry, Biochemistry, Nutrition, and Environment, Faculty of Medicine and Pharmacy, University Hassan II, Casablanca, Morocco; ^3^Central Laboratory, College of Pharmacy, King Saud University, P.O. Box 2457, Riyadh 11451, Saudi Arabia; ^4^Medicinal Aromatic and Poisonous Plants Research Center, College of Pharmacy, King Saud University, P.O. Box 2457, Riyadh 11451, Saudi Arabia; ^5^Elleva Pharma s.r.l. Via Pietro Castellino, 111, CNR Research Area Naples 1, 80131 Naples, Italy; ^6^Research Institute on Terrestrial Ecosystems (IRET), National Research Council, Porano, Italy; ^7^Biology Unit and Medical Research CNESTEN, Rabat 10001, Morocco

## Abstract

**Background:**

*Caralluma europaea* (*C*. *europaea*) is a medicinal plant used in Moroccan popular medicine. *Objective of the Study*. The present work was aimed at identifying the chemical composition and the antioxidant and antiproliferative activities of hydroethanolic and bioactive compound classes of *C*. *europaea*) is a medicinal plant used in Moroccan popular medicine. *Materials and Methods*. The chemical composition was analyzed using HPLC. The antioxidant power was determined using both DPPH and FRAP assays. The antiproliferative activity was effectuated against cancerous cells using WST-1.

**Results:**

The chemical analysis showed the presence of bioactive constituents such as quercetin, myricetin, and hesperetin. The polyphenol and flavonoid contents were estimated at 51.42 mg GA/g and 20.06 mg EQ/g, respectively. The EC_50_ values of FRAP assay of hydroethanolic, flavonoid, saponin, and mucilage extracts were 5.196 mg/ml, 4.537 mg/ml, 3.05 mg/ml, and 6.02 mg/ml, respectively. The obtained IC_50_ values with the DPPH test were 1.628 mg/ml, 1.05 mg/ml, 1.94 mg/ml, and 9.674 mg/ml, respectively. Regarding MDA-MB-231, saponins were highly effective even with the lowest concentration (15.62 *μ*g/ml). The flavonoids decreased the cell viability with IC_50_ values of 43.62 ± 0.06 *μ*g/ml). The flavonoids decreased the cell viability with IC_50_ values of 43.62 ± 0.06 *μ*g/ml). The flavonoids decreased the cell viability with IC_50_ values of 43.62 ± 0.06

**Conclusion:**

The present results suggest that *C*. *europaea*) is a medicinal plant used in Moroccan popular medicine.

## 1. Introduction

Traditional medicine is attracting more interest and its use has increased over the past three decades. According to the WHO, more than 80% of African people still rely on popular medicine for their benefit care including cancer treatment [1]. Nowadays, many pharmaceutical agents have been discovered in medicinal plants. However, the potential of plants as sources for the production of new drugs is largely untapped [2]. Medicinal plants play an important role in folk medicine, and they have been described in worldwide pharmacopoeia [3].

*Caralluma europaea *is a wild species frequently used in Moroccan traditional medicine for its presumed anticancer activity [4]. *C*. *europaea* belongs to the Apocynaceae family, and it is widely spread in Southern Jordan and the North African coast of the Mediterranean Sea [5]. In Morocco, *C*. *europaea* is used in traditional medicine for its anti-inflammatory, antipyretic, antinociceptive, antihyperglycemic, antidiabetic, antitrypanosomal, antiulcer, neuroprotective, antiobesogenic, antiatherosclerotic, and antiparasitic activities [6]. Several scientific studies have confirmed the antioxidant, antimicrobial, and anti-inflammatory activities of *Caralluma europaea* [7–9].

Nowadays, the world witnesses a great revolution in cancer treatment due to the large development of novel pathways of treatment. Although that resistance to classical agents continues to be a major problem in cancer therapies [10]. It was earlier reported that the medicinal plants play an alternative role in breast cancer treatment [11, 12].

A few chemical constituents were reported from *C*. *europaea*, including flavone glycosides such as luteolin 4′-neohesperidoside, luteolin-3′-O-(6′-O-sinapoylglucoside)-4′-O-neohesperidoside, and luteolin-3′-O-(6′-O-feruloylglucoside)-4′-O-neohesperidoside; monoterpenoids; *α*-terpinene; linalool; and terpinolene [5, 8].

The objective of the present study was to screen the phytochemical composition and the antioxidant and antiproliferative effects of both aerial parts hydroethanolic and natural bioactive classes of *C*. *europaea* using the methods described in the next section.

## 2. Materials and Methods

### 2.1. Plant Material

*Caralluma europaea *was collected in February 2016, from the Imouzzer region at the Middle Atlas Mountains, Morocco, using GPS coordinates (33°44′ North, 5°01′ West). The plant was authenticated by Professor Bari Amina, and a voucher specimen, 18I4C001, has been deposited in the herbarium of the Department of Biology, Faculty of Sciences Dhar El Mahraz, Fez University.

### 2.2. Preparation of Hydroethanolic Extract

The aerial parts of the plants were washed with water. After drying in the shade, the material was ground into a fine powder using a grinding apparatus. 10 g of the powder was extracted with 100 ml of hydroethanolic solution (7 : 3 v/v ethanol: distilled water) for 45 min. The mixture was filtered, then concentrated at low temperature, and finally preserved at −80°C until use.

### 2.3. Phytochemical Screening

*C*. *europaea* was screened for potential presence of flavonoids, alkaloids, quinones, catechic tannins, gallic tannins, mucilages, and saponins using the protocols described in [13–16].

### 2.4. Determination of Total Antioxidant Compounds

#### 2.4.1. Total Polyphenol Content

The polyphenolic content (TPC) was determined using Folin-Ciocalteu reagent as previously described in the literature. The results were expressed as mg gallic acid equivalent (mg GAE)/g dry extract [17].

#### 2.4.2. Total Flavonoid Content

The flavonoid content (TFC) was determined according to the method in earlier data. The results were expressed as mg quercetin equivalent (mg QE)/g dry extract [18].

### 2.5. HPLC Analysis

Polyphenolic extract of *Caralluma europaea *was analyzed using high-performance liquid chromatography (Agilent Technologies) equipped with a quaternary pump. The sample was detected by using a UV detector operating at 280 nm. A volume of 10 ml of the extract was injected over a C18 ZORBAX Eclipse Plus (serial number: USUXBO2265) (46 *∗* 150 nm; 5 *μ*m) column, at a flow rate of 1 ml/min, and the column temperature was maintained at 25°C. The mobile phase was composed of acidified water 0.1% (A) and acetonitrile (B) with a total running time of 65 min.

### 2.6. Determination of Antioxidant Activity

#### 2.6.1. Free Radical-Scavenging Ability (DPPH)

The test of DPPH was performed using the methods reported in [19], with slight modifications. A solution of the DPPH was prepared by dissolving 100 ml of methanol and 2.4 mg DPPH. 980 *μ*l of DPPH solution (60 *μ*M) was mixed with 20 *μ*l of an extract with concentrations ranging from 10 to 50 *μ*g ml^−1^. After 2 h of incubation, the absorbance was determined at 517 nm. BHT (10 to 50 *μ*g ml^−1^) was used as a positive control. The percentage of inhibition of DPPH was calculated using the formula given as follows:

(1) $$\begin{array}{c} \text{IP}\left(\%\right)=\left(\frac{\text{A0}-\text{A}}{\text{A0}}\right)*100, \end{array}$$

where IP is the inhibition percentage, A0 is the absorbance of the DPPH solution without extract, and A is the absorbance of the DPPH solution with the extract.

#### 2.6.2. Ferric Reducing Antioxidant Power (FRAP)

The FRAP method determines the capacity of antioxidants to reduce Fe^+3^ to Fe^+2^. 1 ml of extract was dissolved with 2.5 ml of phosphate-buffered saline and 2.5 ml of potassium ferricyanide (1%). After incubation for 20 min at 50°C, 2.5 ml of trichloroacetic acid (10%) was added. The obtained solution was centrifuged for 10 min at 3000 rpm. Then, 2.5 ml of the supernatant was combined with 0.5 ml of FeCl_3_ (0.1%) and 2.5 ml of distilled water. The absorbance was determined spectrophotometrically at 700 nm. Ascorbic acid was considered as a reference [20].

### 2.7. Extraction of Some Bioactive Compound Classes from *Caralluma europaea*

#### 2.7.1. Flavonoids

The powder of *C*. *europaea* (30 g) was macerated with 100 ml of methanol (MeOH) for 72 h at room temperature. The mixture was filtered and concentrated at 60°C. The obtained residue was dissolved in distilled water and then extracted again successively three times with 3 × 30 ml (chloroform, diethyl ether, *n*-hexane, ethyl acetate, and *n*-butanol) to get respective extracts. The flavonoids contained in the last phase were recovered and then subjected to evaporation at 55°C in order to obtain the dry residue [21].

#### 2.7.2. Saponins

10 g of the powder of *C*. *Europaea* was initially defatted with petroleum ether for 2 h. Then, the extraction was carried out with 300 ml of ethanol for 24 h. The obtained extract was fractionated with petroleum ether and distilled water in equal proportions. 150 ml of the aqueous portion was added to *n-*butanol. The *n*-butanol fraction was separated, the remained solution was concentrated, and then the saponins were dried in an oven [22].

#### 2.7.3. Mucilages

10 g of the powder of *C*. *Europaea* was macerated with distilled water for 5 or 6 h, boiled for 30 min, and kept aside for 1 h. The material was filtered through a muslin cloth. Acetone was added to the filtrate in order to precipitate the mucilage. The mucilage was separated and dried at a temperature under 50°C. The dried mucilage was powdered and stored for further use [23].

### 2.8. Evaluation of Antiproliferative Activity

#### 2.8.1. Cell Culture

The antiproliferative activity of natural compounds from the aerial parts of *C*. *europaea* was tested against two human breast cancer cell lines, MDA-MB-231 and MCF-7, provided by Dr. L'Houcine Ouafik (Laboratoire de Transfert d'Oncologie, Marseille). The cells grew in DMEM medium supplemented with glutamine (1%), fetal calf serum (10%), and a mixture of streptomycin/penicillin (1%). Cells were preserved at 37°C.

#### 2.8.2. In Vitro Antiproliferative Activity Assay

The antiproliferative activity was evaluated using WST-1 test (disodium mono{4-[3-(4-iodophenyl)-2-(4-nitrophenyl)-2H-tetrazol]-3-ium-5-yl]benzene-1,3-disulfonate}) [24]. The concentrations tested for their antiproliferative effects ranged from 1.562 *μ*g/ml to 500 *μ*g/ml. Cells of a subconfluent culture were harvested and centrifuged at 100 rpm for 5 min. The prepared cells were seeded in 96-well plates, at a cellular density of 8000 cells/well. Cells were incubated in a humidified atmosphere for 24 h at 37°C (5% CO_2_). After this, 100 *μ*l of fresh medium containing serial concentrations was added to cells for further incubation of 48 h at 37°C.

At the end of the treatment period, the medium was removed, and 10 *μ*l of WST-1 reagent was added to each well. The plates were incubated again (4 h at 37°C). Cell viability was performed by absorbance reading of each well at 450 nm using a Wallac Victor X3 multiplate reader. Untreated cells were considered as a negative control. Mitomycin was considered a positive control. The IC_50_ value responsible for 50% of cell growth inhibition was determined by plotting the inhibition percentage versus the concentrations (*μ*g/ml).

### 2.9. Statistical Analysis

Analysis of the dose-response curves and the IC_50_ values was done using GraphPad Prism 5. Calculation of confidence limits and significance testing were made at the level of *p*=0.05 (*p* value <0.05 was considered significant).

## 3. Results

### 3.1. Qualitative Phytochemical Screening

The results of the phytochemical analysis of hydroethanolic extracts of *C*. *europaea *revealed the presence of flavonoids, catechic tannins, triterpene saponins, mucilages, coumarins, oses, and holosides and the absence of quinones and alkaloids (Table 1).

### 3.2. Total Polyphenol and Flavonoids Content

**Figure 1:**
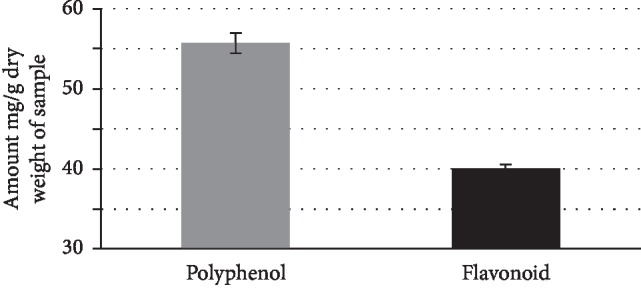
Total polyphenols and flavonoids contents of *C*. *europaea* hydroethanolic extract.

The phenolic content of aerial parts of *C*. *europaea* was determined from the calibration curve (*y* = 1.575*x* + 0.022; *R*^2^ = 0.959). The value was 51.42 ± 0.003 mg gallic acid equivalent/g of extract. Regarding the total flavonoid content (*y* = 0.814*x* + 0.021; *R*^2^ = 0.953), the value was 20.06 ± 0.007 mg QE/g of extract (Figure 1). The obtained results in this study showed a significant level of phenolic compounds contained in aerial parts of *C*. *europaea*.

### 3.3. HPLC Analysis

**Figure 2:**
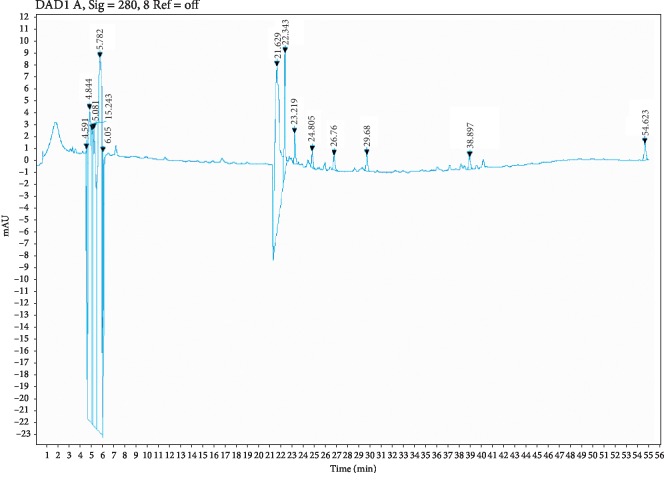
HPLC chromatogram of the main compounds identified in the polyphenolic extract of *C*. *europaea*.

Five compounds were identified in polyphenols extract of *Caralluma europaea* using HPLC: hesperetin (TR = 24.805 min), quercetin (TR = 23.219 min), myricetin (TR = 22.34 min), ferulic acid (TR = 22.629 min), and gallic acid (TR = 6.051 min) (Figure 2).

According to HPLC analysis, the rate of polyphenols contained in *Caralluma europaea* extract ranged from 0.034 to 2.772 *μ*g/ml. The ferulic acid concentration was determined at 2.772 *μ*g/ml, followed by quercetin with 0.77 *μ*g/ml. The concentrations of myricetin, gallic acid, and hesperetin were estimated at 0.350 *μ*g/ml, 0.135 *μ*g/ml, and 0.034 *μ*g/ml, respectively (Table 2).

### 3.4. Evaluation of Antioxidant Activity Using FRAP and DPPH  Assay

The antioxidant activity of *C*. *europaea* extract was determined using two methods: DPPH and FRAP tests. The results vary according to the used methods.

#### 3.4.1. Evaluation of Antioxidant Activity Using FRAP Assay

**Figure 3:**
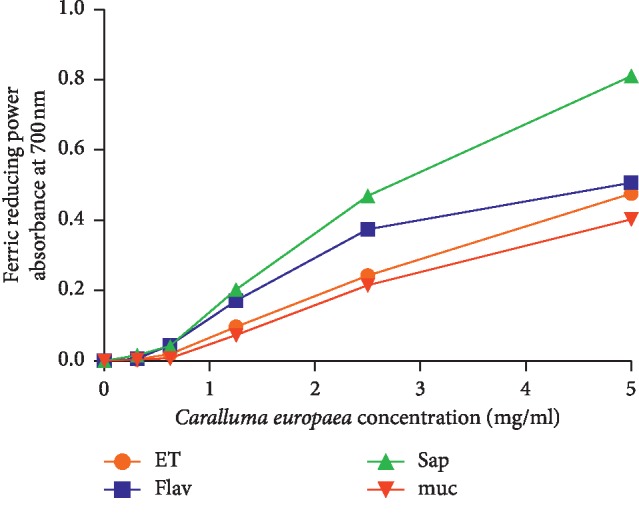
Antioxidant activity of hydroethanolic extract (ET), flavonoids (Flav), saponins (Sap), and mucilages (muc) of *C*. *europaea* using FRAP assay.

**Figure 4:**
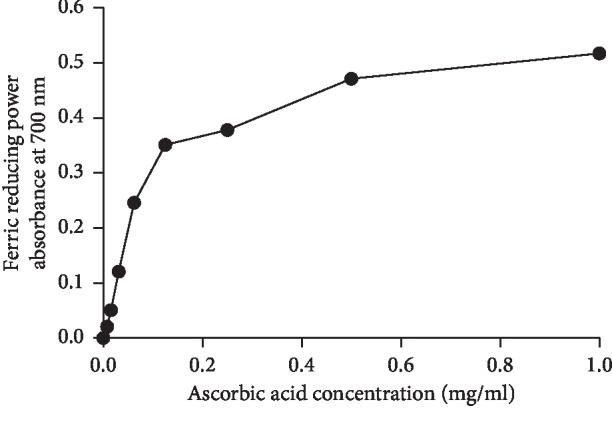
Antioxidant activity of ascorbic acid using FRAP assay.

As indicated in Figure 3, all extracts showed lower antioxidant activities than the standard (ascorbic acid) (Figure 4). The EC_50_ values of hydroethanolic, flavonoids, saponins, and mucilages fractions were 5.196 mg/ml, 4.537 mg/ml, 3.05 mg/ml, and 6.02 mg/ml, respectively.

#### 3.4.2. Evaluation of Antioxidant Activity Using DPPH Assay

**Figure 5:**
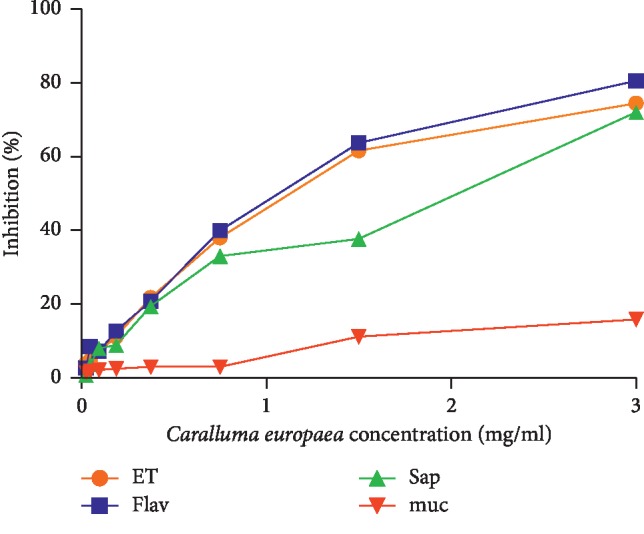
Inhibition percentage of free radicals of hydroethanolic extract (ET), flavonoids (Flav), saponins (Sap), and mucilages (muc) of *C*. *europaea* using DPPH assay.

**Figure 6:**
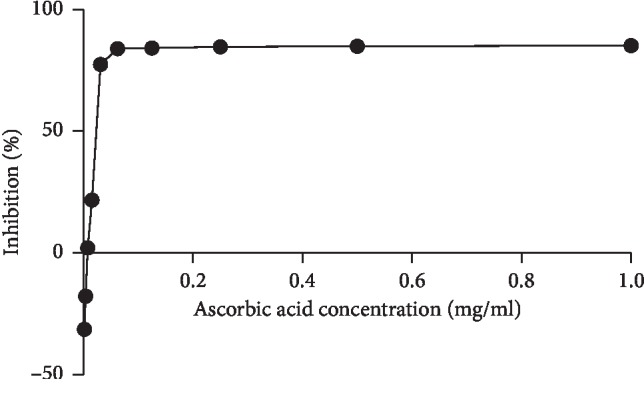
Inhibition percentage of free radicals of ascorbic acid using DPPH assay.

As shown in Figure 5, the percentage of free radical inhibition of hydroethanolic extracts such as flavonoids, saponins, and mucilages was lower than that of ascorbic acid (Figure 6). The antioxidant activity of the flavonoids fraction (IC_50_ = 1.51 mg/ml) was more pronounced compared to the other hydroethanolic extracts (IC_50_ = 1.628 mg/ml), saponins (IC_50_ = 1.94 mg/ml), and mucilages (IC_50_ = 9.674 mg/ml). The statistical analysis showed a significant difference between the IC_50_ values of all tested extracts and that of the ascorbic acid (IC_50_ = 0.23 mg/ml) (*p* value <0.05).

### 3.5. Extraction of Natural Antioxidants from *C*. *europaea*

Genus of *Caralluma* is characterized by the presence of bioactive components such as glycosides and steroids, known for their various biological activities [25–27]. In order to investigate the potential antiproliferative effect of *C*. *europaea* natural compounds, we proceeded with the extraction of saponins, flavonoids, and mucilage. The hydroethanolic extract was also tested for its antiproliferative effect. The extraction yields of various fractions were reported in Table 3.

### 3.6. Antiproliferative Activity

**Figure 7:**
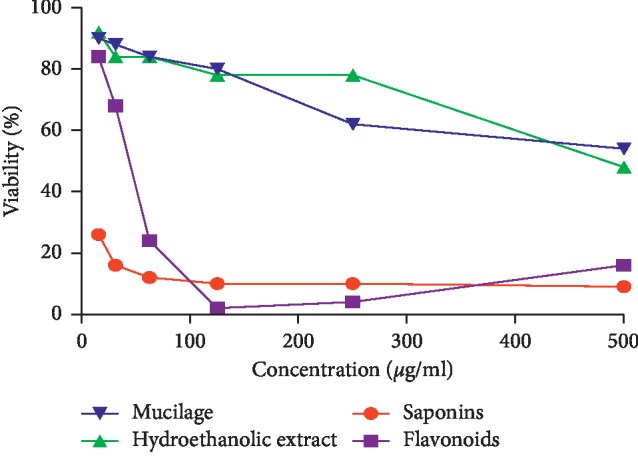
Antiproliferative effect of hydroethanolic extract of *C*. *europaea* and its fractions against MDA-MB-231 cell lines.

The antiproliferative effect of hydroethanolic extract of *C*. *europaea* and its fractions against MDA-MB-231 cell lines was evaluated using the WST-1 assay.  Figure 7 shows the effect of hydroethanolic extract and its fractions at different concentrations (15.6 to 500 *μ*g/ml) on MDA-MB-231 cell viability. Flavonoids fraction showed a remarkable antiproliferative effect with an IC_50_ value of 43.62 ± 0.06 *μ*g/ml. The saponins fraction significantly inhibited cell proliferation with the studied range of concentrations even with the lowest value (15.62 *μ*g/ml). However, hydroethanolic extract and mucilage fraction were ineffective with the current used concentrations.

**Figure 8:**
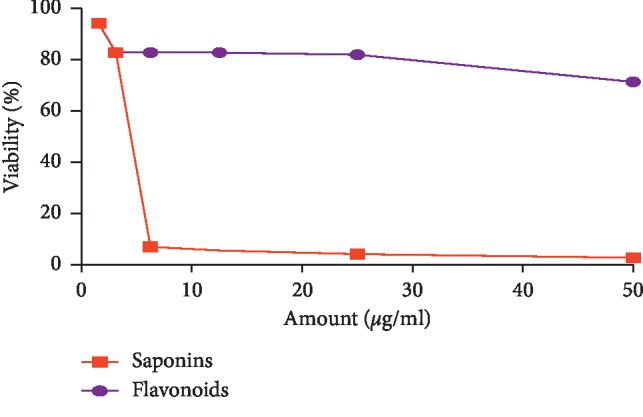
Antiproliferative effect of *C*. *europaea* saponin and flavonoid fractions against MDA-MB-231 cell lines.

**Figure 9:**
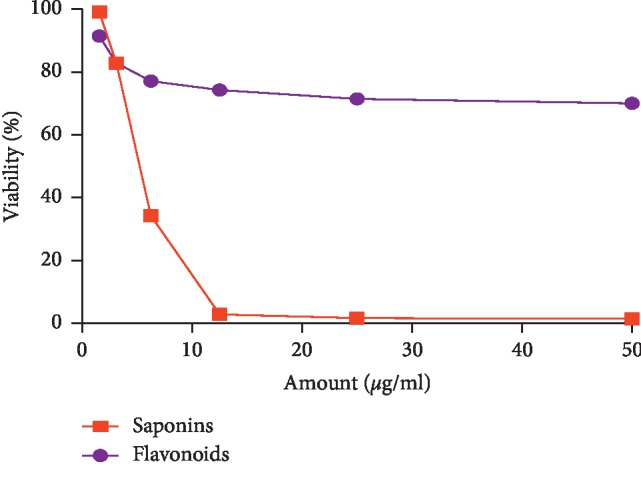
Antiproliferative effect of *C*. *europaea* saponin and flavonoid fractions against MCF7 cell lines.

Saponins fraction of *C*. *europaea* extracts induced an important inhibited effect on both MDA-MB-231 (Figure 8) and MCF cell lines (Figure 9) even with the lowest concentration (1.562 *μ*g/ml). Saponins clearly exhibited significant antiproliferative activity on both cell lines, with IC_50_ values of 5.097 *μ*g/ml and 4.195 *μ*g/ml on MCF7 and MDA-MB-231 cell lines, respectively. Flavonoids were ineffective with the tested range of concentrations. There was no significant difference between the IC_50_ values of saponin fractions on MCF7 no MDA-MB-231 (*p* > 0.05).

## 4. Discussion

Cancer is one of the most lethal diseases worldwide, requiring effective prevention and treatment measures. Therefore, considerable research has been conducted to develop novel natural chemotherapeutic agents. In medicine, plant-derived compounds can be used as traditional, modern drugs, or chemical units for synthetic drugs. Antiproliferative activity models provide data to select extracts of plants with antitumoral potential for future studies [28].

Genus *Caralluma* arouses great interest in scientific fields due to its attractive immunostimulatory and pharmacological activities [29]. These activities could be due to its phytochemicals including glycoside and pregnane steroid [26, 27]. In Morocco, *Caralluma europaea* is widely used in folk medicine for its presumed anticancer activity [30]. However, no scientific data about its potential anticancer activity against cancerous cell lines were reported to the best of our knowledge.

The hydroethanolic extract of *C*. *europaea* was investigated for its phytochemical compounds, the total phenolic and flavonoid contents, and the antioxidant and antiproliferative activities. The qualitative phytochemical study showed the presence of different classes of bioactive secondary metabolites, such as flavonoids, triterpene saponins, oses, and holosides, previously described to have various medicinal activities [31]. Regarding the studied activities, our results were in accordance with those reported for *C*. *sinaica* [32].

It is worth noting that *C*. *europaea* is rich in phenolic compounds; the total phenolic content of *C*. *europaea* hydroethanolic extract was 51.42 ± 0.003 mg GAE/g dry of extract. These values were significantly higher than those of the methanolic and aqueous extract of *C*. *adscendens*, being 21.0 ± 0.59 and 18.8 ± 0.98 mg GAE/g dry extract, respectively [33].

Phenolic compounds are ubiquitous plant metabolites and the major group of compounds that contribute to the antioxidant properties; they can play an important role in the initiation of deleterious free radical actions [34]. Species of the genus *Caralluma* have been known for their richness in phenolic compounds [35, 36].

Our study also demonstrated that the hydroethanolic extract of *C*. *europaea* was rich in flavonoids compounds. The total flavonoids in the hydroethanolic extract of *C*. *europaea* were 20 ± 0.007 mg QE/g dry. This content is still relatively higher compared to methanolic and aqueous extracts of *C*. *adscendens* with values of 3.7 ± 1.16 and 3.1 ± 0.54 mg QE/g dry weight, respectively [33].

Flavonoids are the most important natural phenolic compounds and are thought to be responsible for the antioxidant and the antiproliferative activities of natural products [37, 38]. Many species of *Caralluma* have been known for the presence of interesting amounts of bioactive compounds [39, 40].

Flavonoids and polyphenols are natural antioxidants, known to be very active scavengers of many free radicals [41]. DPPH test is a method for estimating free radical-scavenging activity of antioxidants. The FRAP test determines the ability of these complexes to break the free radical chain by donating an atom of hydrogen [42, 43]. In the current study, the antioxidant activity of *C*. *europaea* hydroethanolic extract was determined using DPPH and FRAP assays. Using the DPPH method, all tested extracts of *C*. *europaea* showed a lower antioxidant power compared to ascorbic acid. The IC_50_ values of hydroethanolic, flavonoids, saponins, and mucilages extracts were 1.628 mg/ml, 1.51 mg/ml, 1.94 mg/ml, and 9.674 mg/ml, respectively, which were significantly lower than that of the ascorbic acid (0.23 mg/ml). Furthermore, the obtained IC_50_ values with FRAP test concerning hydroethanolic, flavonoids, saponins, and mucilages fractions were 5.196 mg/ml, 4.537 mg/ml, 3.05 mg/ml, and 6.02 mg/ml, respectively. These results can be attributed to the phenolic contents of the aerial parts of *C*. *europaea*.

Antiproliferative effect of hydroethanolic extract *C*. *europaea* and its fractions was tested against two human breast cancer cell lines, MDA-MB231 and MCF-7. The tested concentration on the MDA-MB-231 cell line ranged from 15.6 to 500 *μ*g/ml. The flavonoids fraction reduced cell viability in a dose-dependent manner after 48 h of treatment, with an IC_50_ value of 43.62 ± 0.006 *μ*g/ml. Antitumor activity of this extract can be attributed to the identified compounds with the HPLC method, as previously reported, such as hesperetin, quercetin, myricetin, and ferulic acid. With the same range of concentration, saponins fraction seems to be very effective against cell lines; however, mucilage fraction and hydroethanolic extract were ineffective. Antiproliferative effects of saponins and flavonoids fractions were tested again with lower concentrations ranging from 1.562 to 50 *μ*g/ml on MCF7 and MDA-MB- 231 cell lines. Saponins clearly exhibited significant antiproliferative activity on both cell lines, with IC_50_ values of 5.097 *μ*g/ml and 4.195 *μ*g/ml against MCF7 and MDA-MB-231 cell lines, respectively.

Potential antiproliferative and anticancer activities of some species of Caralluma were reported in many earlier research works. The methanolic extract of *Caralluma acutangula* exhibited effects with concentrations varying between 0 and 50 *μ*g/ml on MCF-7 (breast cancer) and HEPG2 (hepatocellular carcinoma) [44]. The methanolic extracts of *Caralluma acutangula* possess an interesting antiproliferative activity on HeLa (cervix cancer), MCF7 (breast cancer), and HEPG2 (hepatocellular carcinoma) cells [45].

Antiproliferative activity of plants may be attributed to the presence of alkaloids, glycosides, saponins, and polyacetylenes [46]. A previous phytochemical study on *Caralluma* species suggested the presence of glycosides and saponins [47]. Saponins are natural compounds that have several pharmacological activities like antiproliferative properties [48, 49], with pronounced effects on malignant tumor cells [9].

Numerous *in vitro* studies have reported the antiproliferative activity of saponins on breast cancer, cervical colon, and hepatic carcinomas. It was reported that several signaling pathways have been identified to be responsible for cell death [49, 50]. The antiproliferative effect of saponins resulted in the activation of different signaling pathways [49], whereas the induction of apoptosis is the most involved pathway [51]. Saponins have an important effect on some experimental tumors, such as estrogen receptor-negative mammary carcinogenesis [52].

In the present research work, flavonoids showed a significant antiproliferative effect with IC_50_ values of 43.62 ± 0.06 *μ*g/ml. This class of molecules possesses a large range of pharmacological activities including antiproliferative activity. The flavonoids constitute the most important group of polyphenolic compounds, which have been investigated for their antiangiogenic, antiproliferative, and antiestrogenic effects [53]. Several works have reported that the flavonoids possess antiestrogenic activity and prevent the development of hormone-dependent cancers [54].

## 5. Conclusion

The findings of the present work revealed that the extract of aerial parts and the natural bioactive classes of *C*. *europaea* exhibit promoting antioxidant and antiproliferative activities. Therefore, *C*. *europaea* may constitute an important source of bioactive compounds which may lead to conceptualizing new effective drugs against cancer as well as diseases due to free radicals.

## Figures and Tables

**Table 1:** Phytochemical screening of *C*. *europaea*.

Compounds	Results
Flavonoids	+
Catechic tannins	++
Triterpenes saponins	++
Mucilages	++
Coumarins	+
Oses and holosides	++
Quinones	−
Alkaloids	−

++: abundantly present; +: present; −: undetected.

**Table 2:** Amounts of the identified compounds in polyphenols extract of *Caralluma europaea* expressed in *μ*g/ml.

Phenolic compounds	Concentrations (*μ*g/ml)
Hesperetin	0.034
Quercetin	0.77
Myricetin	0.350
Ferulic acid	2.772
Gallic acid	0.135

**Table 3:** Yield percentage of *C*. *europaea* bioactive classes.

Compounds	Yields (%)
Flavonoids	1.97
Saponins	2.44
Mucilages	41.3

## Data Availability

The data used to support the findings of this work are included within the article.
